# One Health, climate change, and infectious microbes: a joint effort between AGU and ASM to understand impacts of changing climate and microbes on human well-being across scales

**DOI:** 10.1128/msphere.00035-24

**Published:** 2024-01-31

**Authors:** Antarpreet Jutla, Gabriel M. Filippelli, Katherine D. McMahon, Susannah G. Tringe, Rita R. Colwell, Helen Nguyen, Michael J. Imperiale

**Affiliations:** 1 Department of Environmental Engineering Sciences, GeoHealth and Hydrology Laboratory, University of Florida, Gainesville, Florida, USA; 2 Department of Earth Sciences, Indiana University Purdue University Indianapolis (IUPUI), Indianapolis, Indiana, USA; 3 Department of Civil and Environmental Engineering, University of Wisconsin—Madison, Madison, Wisconsin, USA; 4 Environmental Genomics and Systems Biology, Lawrence Berkeley National Laboratory, Berkeley, California, USA; 5 Institute for Advanced Computer Studies, University of Maryland, College Park, Maryland, USA; 6 Helen Nguyen, Department of Civil and Environmental Engineering, University of Illinois Urbana-Champaign, Urbana, Illinois, USA; 7 Department of Microbiology and Immunology, University of Michigan, Ann Arbor, Michigan, USA; University of Georgia, Athens, Georgia, USA

## EDITORIAL

A historical overview of One Health can be traced to the 400 B.C.E. work of Hippocrates entitled “On Airs, Waters and Places,” in which the philosopher theorized the complex relationship among humans, pathogens, animals, and their surrounding environment. A modern definition of One Health focuses on the interconnectedness of human and animal health and the environment in which people live and work. Although not a novel concept, One Health has gained significance due to factors altering the dynamics of interactions among humans, animals, plants, and the environment, especially after the COVID-19 pandemic. Significant advances have been made in understanding etiological pathways of pathogenesis, but knowledge gaps exist in understanding the effects of climate on the survival and proliferation of pathogens and the impact on the health of human populations.

The life cycles of pathogens and their vectors that are environmentally driven by water (e.g., *Vibrio cholerae, Shigella*, norovirus) and air (e.g., influenza, coronavirus) have been linked to climatic (1), environmental (2), and ecological processes (2). According to the WHO, vector-borne diseases account for more than 17% of all infectious diseases and are responsible for about 700,000 deaths globally every year (3). The vector-borne dengue virus, whose proliferation and transmission are closely linked to ambient temperature fluctuations, causes an estimated 200 to 300 million human cases annually and is an increasing cause of concern worldwide (4). Similarly, waterborne diseases such as cholera, caused by the aquatic bacterium *Vibrio cholerae,* impose a significant public health burden in regions that lack adequate water and sanitation infrastructure (5–7). A recent systematic review of the influenza virus (8) indicates the likelihood of an increase in the global burden of respiratory diseases, which is associated with changes in land use patterns that force wildlife to come into close contact with humans.

Changes in climatic processes, such as warming oceans and ambient temperatures, will likely alter human migration patterns and impact environmental niches of pathogens in ways that enhance the likelihood of interactions of vulnerable human populations with infectious microbes, causing infectious diseases in regions where such outbreaks have not been recorded previously. A global expansion of vibrios (e.g., *Vibrio vulnificus*) from tropical ocean waters to northern latitudes over the last two decades (9, 10) provides one of many compelling cases of changes in water temperatures leading to significant expansion of the ecological niches of potentially harmful microbes.

**Fig 1:**
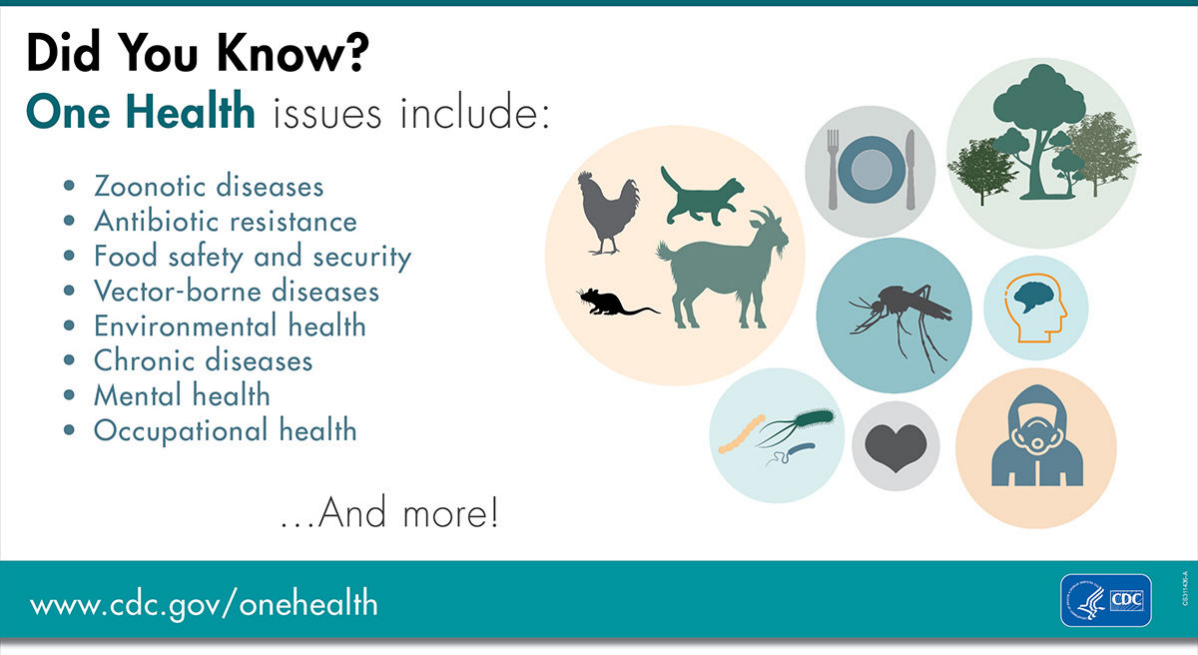
The importance of One Health in the globally connected world (image publicly available from www.cdc.gov).

The relationships among climate change, infectious pathogenic diseases, and humans are highly non-linear and complex, partly because of degrees of freedom in understanding where, when, and how a particular pathogen will likely emerge and impact a given human population. For example, waterborne diseases, such as cholera, are common in regions where climatic processes and extremes are associated with social vulnerabilities. Similarly, chikungunya and dengue, both arboviral diseases, present highly non-linear examples of how the geographical reach of a pathogen can expand with climate change in regions where humans are generally naive to these viruses (11). For microbes that cause infectious diseases in human populations, availability of the epidemiological data remains spotty and such data are only sporadically collected in regions where climate extremes intersect with human vulnerabilities. An additional complication is that microbes are quintessentially a part of Earth’s ecosystem and therefore most cannot be eradicated since host-vector relationships generally have evolved over time. Therefore, it is essential to quantify pathways in which changing climate will impact both microbes and humans alike. One Health (Fig. 1) provides a useful framework to understand such interactions so that a holistic and pre-emptive understanding of human health and disease threats can be defined.

The American Society for Microbiology (ASM) and the American Geophysical Union (AGU) are collaborating to understand the complexities of a changing climate on infectious microbes and how these changes impact human health and well-being. Both scientific societies seek to understand how our changing climate and weather systems will impact human, animal, and plant health. Increasing climatic variability, including extreme weather events, coupled with human-environmental interactions, lead to increased risk of disease outbreaks, including vector-borne (e.g. Zika, dengue, chikungunya, malaria, Rift Valley fever), waterborne (e.g., cholera, dysentery, typhoid), and airborne (e.g. coronavirus, influenza) diseases. While the role of geophysical processes is increasingly appreciated as critical to modulation of microbes, the issues of scale discrepancies limit integration of microbiological understanding of pathogens into large-scale climate and weather patterns. A special collection of papers in *GeoHealth* and *mSphere* will present accumulated knowledge gathered at the interface of climate, weather, and human health, with emphasis on geophysical processes and microbiological functions, information necessary for early warning systems to be developed that can predict risk of diseases (and emergence of pathogens) under current and future changing climate scenarios. Special emphasis will be given to improving our limited arsenal against respiratory infectious pathogens associated with societal determinants and climate modalities.

To submit a manuscript to *GeoHealth*, please use the standard submission portal and select the collection title from the drop-down menu in the Special Collection field of the submission form. To submit your manuscript to *mSphere*, use the standard submission portal and indicate the collection title (One Health, Microbes, and Climate Change) in the cover letter. Queries to the organizers to share your topic proposal and/or abstract prior to submission are encouraged. For presubmission inquiries, please email msphere@asmusa.org or geohealth@agu.org.

mSphere welcomes submissions on:

- Effects of climate change on microbes, microbiomes, microbial community structures, and microbial diversity, particularly in relation to ecosystem health
- Effects of microbes on climate change
- Effects of climate change on microbial pathogens of humans, animals, and plants
- Biogeochemical cycles, nutrient cycling, algal blooms, carbon sequestration, and symbiotic relationships as they relate to climate change

GeoHealth welcomes submissions on:

- Role of microbes in geophysical processes and climate change in different parts of the globe
- Water-, vector-, and airborne diseases related to climate and weather processes
- Respiratory infectious pathogens associated with climate modalities
- Microbial diversity and impact on Earth-climate models: assimilation of data, use of machine learning and other advanced algorithms

## EDITOR'S NOTE

This article is co-published in *mSphere* and *Eos*. The articles are identical except for minor stylistic differences in keeping with each publication's style. Either citation can be used when citing this article.
